# Heterotopic ossification after alloplastic temporomandibular joint replacement: a case cohort study

**DOI:** 10.1186/s12891-022-05582-5

**Published:** 2022-07-04

**Authors:** Ruoyi Ding, Chuan Lu, Jieyun Zhao, Dongmei He

**Affiliations:** 1 Department of Oral Surgery, Ninth People’s Hospital, Shanghai Jiao Tong University School of Medicine, 639 Zhi Zao Ju Road, Huang Pu District, Shanghai, 200011 China; 2 Shanghai Key Laboratory of Stomatology & Shanghai Research Institute of Stomatology, No. 639 Zhi Zao Ju Road, Huang Pu District, Shanghai, 200011 China; 3 National Clinical Research Center of Stomatology, No. 639 Zhi Zao Ju Road, Huang Pu District, Shanghai, 200011 China

**Keywords:** Temporomandibular joint, Alloplastic joint replacement, Complication, Heterotopic ossification

## Abstract

**Background:**

Heterotopic ossification (HO) is one of the serious complications leading to the failure of alloplastic temporomandibular joint replacement (TJR). However, there was few research on its exact incidence and occurrence. Severe HO might result in pain and limited mouth opening after surgery. Therefore, it is necessary to clarify its clinical and imaging manifestations. The purpose of this study was to study the occurrence and classify HO after the alloplastic TJR.

**Method:**

Patients who underwent standard TJR (Zimmer Biomet stock prostheses or Chinese stock prostheses) with fat graft and at least 1-year-follow-up were included. HO was classified into 4 types according to postoperative computed tomography (CT) scans. Type and occurrence in different TMJ disease were compared. Joint space within 1 week after operation was measured and compared between HO and non-HO TJRs. Maximum incisal opening (MIO), pain, and quality of life (QoL) were recorded and their relevance with HO was analyzed statistically.

**Result:**

81cases with 101 joints were included in the study. The mean follow-up time was 22.9 months (12 ~ 56 months). Among the 48 joints, 27 (56.3%) were type I (bone islands); 16 (33.3%) were type II (bone spurs from the mandibular ramus); 3 (6.3%) were type III (bone spurs from the fossa); and 2 (4.2%) were type IV (bone spurs from both the mandibular ramus and fossa). In HO patients, joint space in type IV was smaller than the other 3 types. Pain scores in HO were significantly greater than non-HO patients before and after operations (*p* < 0.05). 1 patient in Type IV HO developed ankylosis and had prosthesis revision which accounted for 2.1% in HO patients and 1.0% in all TJR patients.

**Conclusion:**

HO after alloplastic TJR with fat graft was not severe except for type IV, which was easy to cause ankylosis. Preserving sufficient TJR space was important for ankylosis prevention.

## Introduction

Temporomandibular joint (TMJ) disease can cause swelling or pain, joint snapping, mandibular asymmetry and limited or deflected mouth opening [1]. The first choice for treatment was conservative ways such as exercises, occlusal splint therapy etc. However, when comes to the late stage of the disease, the alloplastic temporomandibular joint replacement (TJR) would be a more effective therapy [2, 3]. Alloplastic TJR is an important treatment for advanced diseases of TMJ such as tumor, osteoarthritis and ankylosis. After more than 30 years of clinical application and follow-up, it can significantly increase patients' mouth opening and joint function, reduce pain, and improve the quality of life [4, 5]. However, there are still some complications after TJR, such as infection, heterotopic ossification (HO), which may require prosthesis revision or replacement [6]. HO was the secondly most common cause after infection in prosthesis revision or replacement, especially when ankylosis and/or severe pain happened [7].

HO is the appearance of mature bone tissue in soft tissues including muscles, tendons, or articular capsules [7]. The incidence of HO in total hip/knee arthroplasty (THA/TKA) ranges from 15 to 90% [8, 9]. However, there are few reports on the occurrence of the HO after TMJ TJR, as well as its affection on patients.

Based on clinical and CT follow-up of patients underwent standard alloplastic TJR, this study analyzed the occurrence of HO and its clinical relevance according to the proposed classification. By measuring postoperative TJR joint space (the shortest distance between the stump of the mandibular ramus and the glenoid fossa on the coronal reconstruction of CT scan) in HO and non-HO joints, the possible affective reasons were clarified.

## Patients and methods

### Study design

This study was a retrospective study which was approved by the hospital ethical board (SH9H-2021-T111-2) and followed the guidelines of the Declaration of Helsinki. Patients who underwent standard alloplastic TJR in our department from June 2015 to December 2020 were enrolled. The inclusion criteria were as follows: (1) clinical and CT examination pre-and post-operation and at least 12 months follow-up; (2) operated by the same surgical method and using fat graft to fill dead space; (3) using Zimmer Biomet stock prosthesis or Chinese stock prosthesis composed of ultra-high-molecular-weight polyethylene (UHMWPE) fossa prosthesis and Cr-Co-Mb mandibular prosthesis. Exclusion criteria were: (1) preserving the attachment of lateral pterygoid muscle; (2) postoperative infection; (3) TJR before.

### Surgical procedure

Pre-auricular and retromandibular incisions were used to expose the glenoid fossa, condyle and lateral surface of the mandibular ramus. The condyle was cut at the neck and removed after lateral pterygoid muscle (LPM) detachment. Instead of discectomy, the discs without severe deformity in osteoarthritis cases were salvaged and pushed medial to the condylar prostheses without dissection of the bilaminar zone. This avoided excessive bleeding [10]. Bone graft from the trimmed articular eminence or condylar neck was fitted into the deep fossa to achieve a flat surface in combination with the residual eminence. Bone repair was in case of a shallow fossa. After trimming the articular eminence, the ramus stump and lateral side of the mandibular ramus, the fossa prosthesis was placed and secured with at least 4 screws. Dressings and gloves were changed after intermaxillary fixation. The condylar prosthesis was installed with the head seat superior-posteriorly in the fossa. Subcutaneous free fat harvested from either the retromandibular or abdominal periumbilical incision was filled around the joint space. A drain was placed into the incision.

### CT evaluation and HO classification

CT scan was performed within 1 week after operation and during at least 1 year follow-up. The parameters of the 64-row-dual-source CT scanner (Somatom Definition Flash; Siemens, Forchheim, Netherlands) were continuous scanning with a layer thickness of 0.625 mm, 120 kV, and tube electricity current of 284 mAs. The image was saved in DICOM format and then imported into Proplan CMF 3.0 (Materialise, Leuven, Belgium). The bone window was selected. Then the coronal plane was reconstructed for evaluation.

**Fig. 1:**
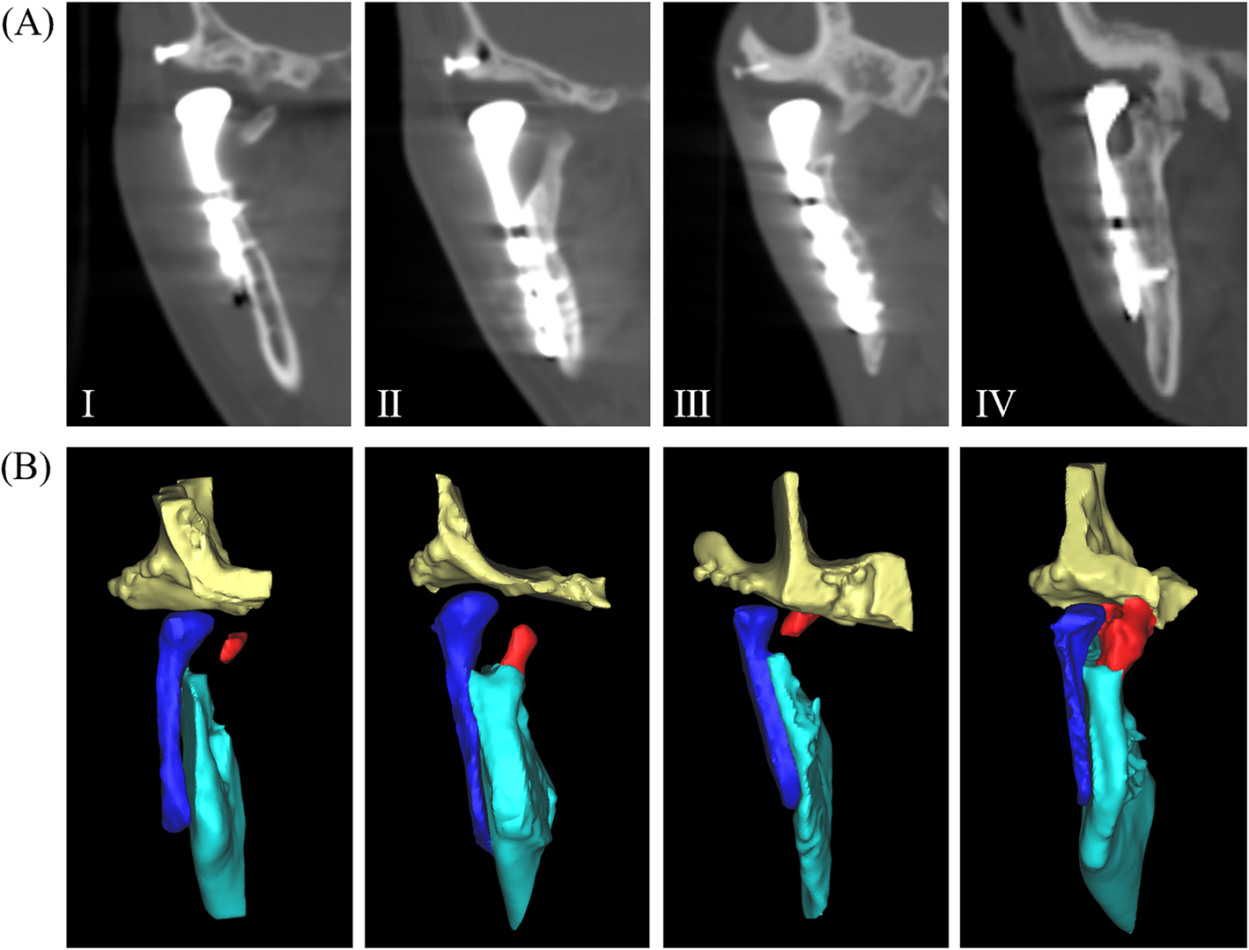
The classification of heterotopic ossification (HO) after alloplastic temporomandibular joint replacement. **A** coronal reconstruction of CT, **B** three-dimensional reconstruction of CT. Red, HO; dark blue, prosthesis; blue, mandible; yellow, skull base

According to Brooker’s [8, 11] classification (Table 1) of HO after TKA in 1986 and Turlington-Durr grading system [12] of TMJ TJR in 1993, we classified HO into 4 types (Fig. 1).

**Table 1:** Brooker's classification of HO after TKA in 1986

Classification	Description
Grade I	Islands of bone within the soft tissues about the hip
Grade II	Bone spurs from the pelvis or proximal end of the femur, leaving at least 1 cm between opposing bone surfaces
Grade III	Bone spurs originating from the pelvis or proximal end of the femur, reducing the space between opposing bone surfaces to less than 1 cm
Grade IV	Apparent bone ankylosis of the hip

*HO* heterotopic ossification, *TKA* total knee arthroplasty

Type I, bone islands within the medial soft tissue of the condylar prosthesis.

Type II, bone spurs from the mandibular ramus stump.

Type III, bone spurs from the medial side of the fossa.

Type IV, bone spurs from both the mandibular ramus stump and fossa.

**Fig. 2:**
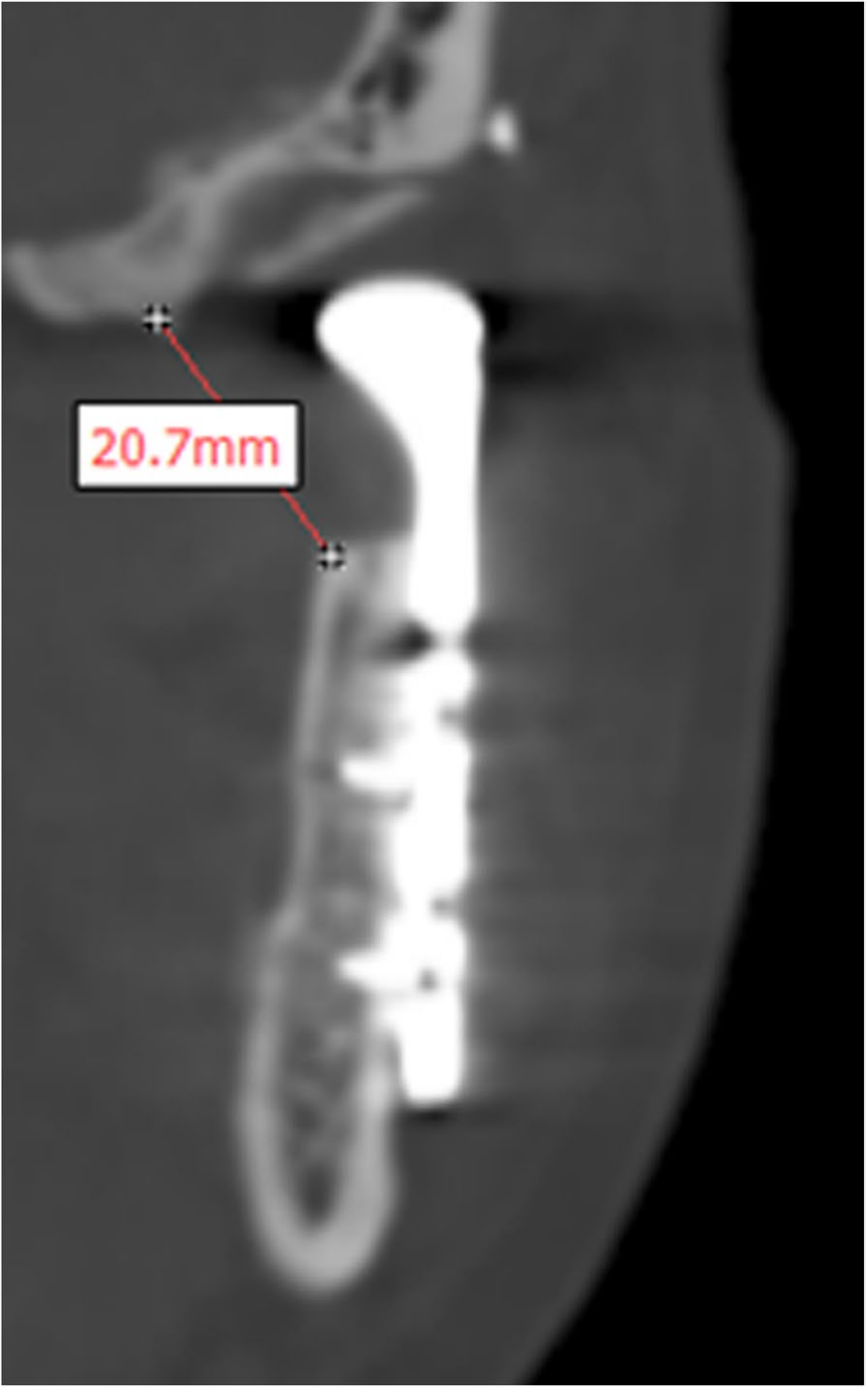
Joint space measurement in the CT coronal reconstruction 1 week after operation

TJR joint space was measured from the coronal reconstruction of CT scan within 1 week after operation. The shortest distance between the stump of the mandibular ramus and the glenoid fossa was recorded by the software tools in millimeter with an accuracy of 0.1 mm (Fig. 2).

## Clinical evaluation

The maximum incisal opening (MIO) was measured and recorded in mm. Visual analogue scale (VAS) was used to evaluate patient-reported pain from 0 to 10 (a continuous scale comprised of a horizontal line which was 10 centimeters in length; 0, no pain; 10 worst pain, a higher score indicates greater pain intensity) [13]. Patients were asked to select a point on a line to report current pain intensity or pain intensity in the last 24 hours. Dimitroulis questionnaire [14] for quality of life (Qol) evaluation was adopted, which involved 8 questions on pain, diet speech, social activities, entertainment, subjective evaluation of disease state, anxiety severity, and an overall evaluation. Each question was followed by 5 options and had a score ranging from 1~5. The total score was calculated, 8–10 points was considered excellent, 11–14 points good, 15–19 points medium, and ≥ 20 points bad.

### Statistical analysis

IBM SPSS software package, version 24.0 (IBM Corp., Armonk, NY, USA) was carried out for statistical analysis. T test was used for comparing the significance of differences in MIO, VAS-pain scores and QoL scores within and between HO and non-HO patients before and after operation. Chi-square test was used to compare the incidence of HO among different diseases. One-way analysis of variance (ANOVA) was used to analyze the differences in joint space among the 4 HO types. An α level ≤ 0.05 was considered significant.

## Results

81 cases with 101 TJRs were included in the study. Among them, there were 20 males and 61 females with an average age of 47.2 ± 14.1 years (range, 18–84 years). 20 cases had bilateral TJR, and 61 cases were unilateral. The diseases included ankylosis in 26 cases with 37 joints, osteoarthritis in 42 cases with 51 joints, TMJ tumor and tumor like lesions (osteochondroma, synovial chondromatosis and pigmented villonodular synovitis) in 13 cases with 13 joints. The mean follow-up period was 22.9 ± 11.3 months (range, 12–56 months).

48 TJRs had various degree of HO, which accounted for 47.5% (Table 2). There were 27 joints (56.3%) in type I, 16 joints (33.3%) in type II, 3 joints (6.3%) in type III and 2 joints (4.2%) in type IV. The incidence of HO in different disease was 43.2% in ankylosis (16/37), 51.0% in osteoarthritis (26/51), and 46.2% in TMJ tumors (6/13). There was no statistical differences among the three diseases (*p* = 0.76, Table 3). After operation, the incidence of Types I and IV HO was the highest in ankylosis patients; Type II was more likely to form in osteoarthritis patients and Type III in tumor patients. However, there were no significant differences among the 3 diseases in Type I and II HO. Due to the small number of Type III and IV patients, statistics cannot be made.

**Table 2:** Incidence, classification of HO and joint space measurement

Classification	Number of joints (%)	Joint space after operation
HO joints (%)	48 (47.5%)	18.3 ± 4.3^#^
Type I	27 (56.3%)	19.3 ± 3.3^*^
Type II	16 (33.3%)	17.4 ± 4.6^*^
Type III	3 (6.3%)	19.6 ± 4.0
Type IV	2 (4.2%)	9.1 ± 1.1
Non-HO joints	53 (52.5%)	19.4 ± 4.2^#^
Total	101 (100%)	

*HO* heterotopic ossification

^#^*p* = 0.31,^*^p = 0.89

**Table 3:** HO distribution among different TMJ disease

	Ankylosis	Osteoarthritis	Tumors	Total
HO	16 (43.2%)^#^	26 (51.0%)^#^	6 (46.2%)^#^	48 (47.5%)
Type I	10 (62.5%)*	14 (53.8%)*	3 (50.0%)*	27 (56.3%)
Type II	4 (25.0%)^@^	11 (42.3%)^@^	1 (16.7%)^@^	16 (33.3%)
Type III	1 (6.3%)	0 (0.0%)	2 (33.3%)	3 (6.3%)
Type IV	1 (6.3%)	1 (3.8%)	0 (0.0%)	2 (4.2%)
Non-HO	21 (56.8%)^	25 (49.0%)^	7 (53.8%)^	53 (52.5%)
Total	37 (100%)	51 (100%)	13 (100%)	101 (100%)

*HO* heterotopic ossification, *TMJ* temporomandibular joint

^#^*p* = 0.76, *p = 0.81, ^@^*p* = 0.32, ^*p* = 0.77

The mean TJR joint space of HO and non-HO patients were 19.4 ± 4.3 mm and 19.2 ± 4.4 mm respectively. There was no statistical difference between them (*p* = 0.31, Table 2). But in Type IV HO, the joint space was smaller (9.1 ± 1.1 mm) compared with Type I (19.3 ± 3.5 mm), Type II (17.4 ± 4.4 mm), and Type III (19.2 ± 4.0 mm) HO patients. But due to the small number of Type IV patients, statistics cannot be made.

Clinical follow-up showed that both HO and non-HO patients had their MIO, pain and QoL improved after TJR (*p* < 0.01, Table 4). Pain scores in HO patients were significantly higher than that in non-HO patients both before and after operations (*p* < 0.05). There were no significant differences of MIO and QoL between HO and non-HO patients both before and after operations (*p* > 0.05). In Type I-III HO patients, MIO and pain were improved after operation, but not in Type IV HO patients (Table 5). One patient developed ankylosis and had prosthesis revision to improve MIO and pain. The prosthesis revision rate was 2.1% in HO patients and 1.0% in all TJR patients.

**Table 4:** Clinical follow-up of patients with and without HO

	HO		Non-HO		P1	P2	P3	P4
	Pre-Op	Post-Op	Pre-Op	Post-Op				
MIO	24.1 ± 11.8	34.7 ± 6.8	22.0 ± 14.7	35.7 ± 7.8	0.00	0.00	0.49	0.54
Pain	4.4 ± 2.9	1.8 ± 2.3	3.0 ± 2.8	0.9 ± 1.1	0.00	0.00	0.03	0.04
QoL	19.4 ± 6.0	13.8 ± 4.9	18.6 ± 5.8	12.2 ± 3.0	0.00	0.00	0.55	0.09

*HO* heterotopic ossification, *Op* operation, *MIO* maximal incisal opening, *QoL* quality of life

*P1* pre- and post-operative value comparison of HO patients, *P2* pre- and post-operative value comparison of non-HO patients, *P3* pre-operative value comparison between HO-patients and non-HO patients, *P4* post-operative value comparison between HO-patients and non-HO patients

**Table 5:** Clinical follow-up of patients with different types of HO

HO	MIO (mm)		*P* value	Pain-VAS		*P* value
	Pre-Op	Post-Op		Pre-Op	Post-Op	
Type I	26.3 ± 11.9	36.2 ± 5.9	0.00	4.9 ± 2.9	1.0 ± 1.3	0.00
Type II	21.4 ± 11.9	34.1 ± 6.6	0.00	3.9 ± 2.7	2.6 ± 2.8	0.10
Type III	28.0 ± 10.6	35.8 ± 2.1	/	1.7 ± 2.4	0.5 ± 0.7	/
Type IV	19.0 ± 11.0	23.0 ± 8.0	/	3.5 ± 1.5	3.8 ± 2.3	/

*HO* heterotopic ossification, *MIO* maximum incisor opening, *VAS* visual analogue scale, *Op* operation

## Discussion

Temporomandibular joint diseases can seriously affect the patients’ joint function and quality of life. With the raise of age, the abnormal joint anatomy, occlusal disorders, high mental pressure and the bruxism caused by it, etc., TMJ diseases are getting more and more popular [15–18]. In addition, temporomandibular joint diseases are more common in women [1]. Therefore, the number of female patients enrolled in this study was much higher than that of male patients. The treatment methods of TMJ diseases include non-invasive treatment such as physical therapy, occlusal adjustment or medication, etc.), minimally invasive treatment (joint injection of platelet rich fibrin, arthroscopy, etc.) and invasive treatment (disc repositioning and alloplastic TJR, etc.) [19, 20].

Alloplastic TJR has been widely used as a major method of TMJ reconstruction since material and design improvements in the 1990s [4]. Although it acquired good results during long-term follow-ups, infection and HO are the main causes of prosthesis revision or replacement [21]. There were few reports on the TMJ HO after alloplastic TJR compared to the one from orthopedics, especially after Wolford [22] proposed using abdominal periumbilical fat to fill the dead space around the joint prosthesis in 1997. The incidence of HO has been significantly reduced than before [23]. In this study, we used subcutaneous or abdominal periumbilical fat for TJR. CT follow-up showed that HO was happened only in the medial side of the prosthesis.

In 1993, Turlington and Durr proposed TMJ HO grading system according to Brooker’s THA HO classification [12]. It is as follows: Grade 0: no bone islands visible; Grade 1: Islands of bone visible within soft tissue around joint; Grade 2: Periarticular bone formation; Grade 3: Apparent bony ankylosis. Grades 1, 2, and 3 were further classified as symptomatic (S) and asymptomatic (A). Symptomatic ossification includes severe pain, decreased interincisal opening (15 mm or less), closed locking of the jaw, or decreased lateral or protrusive movement. In this study, we referred the above classification and described the HO according to its location based on coronal CT reconstruction. Our results showed that most HO was from Type I, bone islands within the medial soft tissue of the condylar prosthesis (56.3%), and Type II, bone spurs from the mandibular ramus stump. There were only 3 patients in Type III (6.3%) and 2 patients in Type IV (4.2%). By analyzing the relevance between HO type and clinical signs and symptoms, we found that Type I ~ III were mild that did not cause mouth opening limitation or pain during follow-ups. Whereas Type IV was the most severe HO which was prone to cause ankylosis and affected MIO and pain. This was similar to the report after THA, although the incidence of small-volume HO can be up to 50%, only 10–20% of the patients have significant discomfort due to the severely affected joint mobility [24, 25]. In our study, Type IV was rare and only accounted for 4.2% of HO. Only 1 patient in Type IV had prosthesis revised to improve MIO and relieve pain. The prosthesis revision rate was 2.1% in HO patients and 1.0% in all TJR patients, which was similar to the Bach's meta-analysis (1.42%) [26].

The location of HO indicates the cause of its formation. HO in Type II-IV developed from the osteotomy plane. This was related to the integrity of cortical bone and the exposure of cancellous bone. By measuring and comparing the joint space within 1 week after operation for the patients developed to HO, we found that type IV HO had significantly smaller joint space than the other 3 types, which is a risk factor for ankylosis. Studies have shown that when the gap between bone stumps was less than 10 mm, ankylosis was more likely to occur [27]. When the bone defect is larger than critical-sized defect (CSD), osseous connection will not form. Animal experiments on dogs with similar mandibular size as human showed that CSD is about 15 mm [28, 29]. At present, there is no requirement for the minimum TJR space when implanting the prosthesis. Although the position of condylectomy is suggested to be at the level of sigmoid notch with removal of coronoid process, for patients with short mandibular ramus, sacrificing a certain joint space to provide sufficient bone support for the mandibular prosthesis may increase the risk of ankylosis. Therefore, it is recommended to use a customized prosthesis with mandibular body extension instead of a standard prosthesis which only fixes the mandibular ramus.

Type I HO was bone island formed medially to the prosthesis. Studies on the etiology of HO have shown that surgical trauma can cause inflammation and activate mesenchymal stem cells in tissues, or scattered from the osteotomy plane, thus differentiating into osteogenesis [30–32]. In addition, the tension of masticatory muscle can also lead to the bone formation [33, 34]. Tendons and ligaments may ossified [35] and disc ossification was also reported after operation [36]. But the mechanism of HO formation after alloplastic TJR remains to be further investigated. Types I and IV HO were more likely to form in ankylosis, Type II in osteoarthritis and Type III in TMJ tumor. In addition, we found that pain scores were significantly higher in HO patients than non-HO patients both before and after operation. High pain scores may reflect local inflammation around the joint which may affect bone metabolism and lead to the occurrence of HO after surgery [37–39]. HO is also a major cause of postoperative pain. So it is important to prevent HO after TJR surgery.

Except periarticular autogenous fat grafting to prevent HO, postoperative radiotherapy and oral non-steroidal anti-inflammatory drugs (NSAIDs) such as indomethacin, celecoxib and bisphosphonates are also reported effective [24]. In 1993, Durr et al. [12] found that early postoperative administration of 10 Gy radiation five times a day could prevent 67% of HO after TJR with a history of ankylosis. Jensen et al. [40] also demonstrated that postoperative radiotherapy could prevent long term HO reformation in 50% of the TJR patients. NSAIDs is another method to prevent HO and relief pain by inhibiting the synthesis of inflammatory factor Prostaglandin E2 (PGE2) [41]. Bhatt et al. [42] found that indomethacin was effective in the prevention of HO after recurrent ankylosis. Naylor et al. [43] found that celecoxib significantly reduced the incidence of HO from 14.3% to 4.3% after THA. Ouyang et al. [44] proved that celecoxib was effective in post-traumatic TMJ HO in animal models. These methods above can be prophylactic used in high-risk patients such as ankylosing spondylitis, hypertrophic osteoarthritis, and recurrent HO.

From the above possible HO formed reasons, we propose several methods which may help HO prevention: During operation, maintain at least 10 mm joint space, using bone wax to seal the osteotomy plane and give sufficient fat graft for dead space filling; After operation, using NSAIDS or radiotherapy for recurrent patients. This study involved patients with more than 1 year follow-up. HO can be shown from CT scan 3 months after operation and matured without change around 6–12 months after operation. In the future, quantitative measurement of HO and long-term follow-up can be taken to observe HO development and the relationship with inflammation. The incidence of HO in the customized TMJ prosthesis with different materials will be studied and compared with the standard TJR.

In conclusion, HO happened in various degrees after alloplastic TJR with fat graft. Most of which have little impact on patients' mouth opening or quality of life. However, type IV HO is prone to cause ankylosis, which need surgical removal to improve MIO and pain relief. Sufficient TJR space may reduce the risk of ankylosis.

## Data Availability

The data collected and analyzed in the current study are not publicly available due to ethical restrictions, but are available from the corresponding author upon reasonable request.

## Abbreviations

CSD: Critical-sized defect
CT: Computed tomography
HO: Heterotopic ossification
LPM: Lateral pterygoid muscle
MIO: Maximum mouth opening
QoL: Quality of life
THA/TKA: Total hip/knee arthroplasty
TJR: Temporomandibular joint replacement
TMJ: Temporomandibular joint
UHMWPE: Ultra-high-molecular-weight polyethylene
VAS: Visual analogue scale
